# Tear Protein Alteration in Dogs with Keratoconjunctivitis Sicca

**DOI:** 10.3390/ani16020160

**Published:** 2026-01-06

**Authors:** Takuya Yogo, Kunihiko Terakado, Kinya Katayama

**Affiliations:** 1 Laboratory of Veterinary Surgery, Nippon Veterinary and Life Science University, Musashino 180-8602, Tokyo, Japan; 2 Petemo Animal Medical Center Sagamihara, Sagamihara 252-0344, Kanagawa, Japan; 3 Laboratory of Biomolecular Chemistry, Nippon Veterinary and Life Science University, Musashino 180-8602, Tokyo, Japan

**Keywords:** canine dry eye, two-dimensional electrophoresis, MALDI-TOF MS, tear film biomarkers

## Abstract

Keratoconjunctivitis sicca (KCS) in dogs is an immune-mediated disorder characterized by aqueous tear deficiency, ocular surface inflammation, and risk of vision loss. Although tear quantity is routinely evaluated using the Schirmer tear test (STT), the accompanying qualitative alterations in tear protein composition remain poorly understood. In this exploratory study, we identified and characterized qualitatively differentially expressed tear proteins in samples collected from seven Beagle dogs with KCS and five healthy Beagles. Samples were collected using filter paper, extracted in phosphate-buffered saline, concentrated by trichloroacetic acid precipitation, and then separated via two-dimensional electrophoresis. Differential protein spots were identified by MALDI-TOF-MS-based peptide mass fingerprinting. Total protein concentrations were determined by measuring UV absorbance at 280 nm and were found to be significantly higher in dogs with KCS (30.7 ± 13.5 mg/mL) than in healthy dogs (11.5 ± 1.8 mg/mL, *p* < 0.05). Five proteins were identified as differentially expressed: serum albumin, lactotransferrin isoform 1, immunoglobulin gamma heavy chain C, major allergen Can f 1, and lysozyme C. High-molecular-weight proteins were upregulated in KCS, whereas low-molecular-weight proteins (<10 kDa, proline-rich protein-like components) were markedly reduced or absent. These compositional shifts suggest that KCS alters both the quantity and qualitative integrity of the tear proteosome, reflecting impaired tear film homeostasis and diminished ocular surface defense. The results support the potential utility of the tear proteome as a source of diagnostic and therapeutic biomarkers in canine KCS.

## Simple Summary

Keratoconjunctivitis sicca (KCS) is a common cause of dry eye in dogs and leads to chronic ocular surface inflammation. While reduced tear production is a well-known feature of KCS, changes in tear protein composition are less well understood. Beagles are not considered a breed highly predisposed to KCS and typically develop the disease later in life; therefore, this study focused on Beagles to minimize breed-related effects. We compared tear proteins from healthy Beagles and Beagles with naturally occurring KCS using proteomic analysis. Dogs with KCS showed increased levels of high-molecular-weight proteins, such as serum albumin and immune-related proteins, while small protective proteins were reduced. These findings suggest that KCS not only reduces tear volume but also alters the protective protein components of tears, potentially compromising ocular defense. This pilot study highlights the value of tear proteomics in advancing our understanding of KCS pathology and in identifying potential biomarkers for diagnosis and therapeutic monitoring.

## 1. Introduction

Keratoconjunctivitis sicca (KCS), classified as aqueous-deficient dry eye (ADDE) in humans, represents one of the most clinically relevant subtypes of dry eye disease (DED). Tear hyperosmolarity and inflammation are recognized as central mechanisms in DED pathogenesis, triggering protease release, cytokine upregulation, and inflammatory cell infiltration that together lead to epithelial damage and tear film instability [1]. In advanced KCS, subsurface vesicles within corneal epithelial cells are lost, and age-related lacrimal keratoconjunctivitis is characterized by dysfunction of regulatory T cells [1]. These cellular and immunologic changes underpin the chronic ocular surface inflammation seen in both humans and dogs.

From a therapeutic standpoint, several ophthalmic medications—including topical cyclosporine (0.05–0.1%) and sodium hyaluronate (0.1–0.3%)—have been approved for the management of DED [1]. Although these treatments improve symptoms, a persistent need remains for objective biomarkers capable of distinguishing disease subtypes and monitoring treatment response. For example, the point-of-care assay for matrix metalloproteinase-9 (MMP-9) represents an early step toward biomarker-driven diagnosis [1], underscoring the growing clinical emphasis on molecular profiling in ocular surface disease.

Canine KCS shares essential immunopathological features with human Sjögren’s syndrome, positioning the dog as a valuable comparative model for autoimmune dry eye [2,3]. At the same time, KCS is a major cause of ocular surface morbidity in dogs, frequently resulting in chronic discomfort, corneal ulceration, and vision loss. These dual aspects—its translational relevance and its clinical burden—together highlight the need for deeper molecular characterization of the disease, particularly to support earlier diagnosis and more precise therapeutic monitoring in veterinary practice.

Mass spectrometry-based proteomics has fundamentally expanded our understanding of tear composition and ocular surface homeostasis. Using these high-resolution analytical platforms, researchers have identified more than 1300 human tear proteins, revealing the biochemical complexity underlying tear film stability. Jung et al. reported 1448 proteins in lacrimal fluid, 849 of which were also detected in tears [4]. Approximately 39% of tear proteins are enzymes—including dehydrogenases, phosphatases, and kinases—that participate in metabolic and signaling pathways [5], while other abundant proteins such as proline-rich protein 4 (PRR4) contribute to ocular surface lubrication and protection [6]. Major protein families defined through proteomic profiling include immunoglobulins, serpins, and 14-3-3 proteins [5]. Collectively, these findings demonstrate the value of tear proteomics as a minimally invasive platform for biomarker discovery and for elucidating ocular surface disease mechanisms [7].

In dogs, proteomic studies have identified between 125 and 505 tear proteins involved in ocular defense, metabolism, and immune regulation [8,9]. These findings highlight the biochemical complexity of the canine tear film. Recent MALDI-TOF MS and LC–MS/MS studies have demonstrated that tear protein composition varies under both physiological and pathological conditions in dogs [9,10,11].

Notably, ocular and systemic diseases can induce measurable alterations in the canine tear proteome. Using two-dimensional electrophoresis (2DE) combined with MALDI-TOF MS, Winiarczyk et al. reported disease-associated remodeling of tear proteins in diabetic dogs with and without retinopathy, suggesting that retinal pathology can be reflected in tear film composition [11].

Technical advances have also expanded the analytical scope of tear proteomics. Recent advances in targeted mass spectrometry have enabled reliable detection of multiple pro-inflammatory cytokines in canine tears, even in clinically healthy Beagles [12]. This methodological progress underscores the expanding potential of tear-based analyses for assessing ocular surface inflammation.

However, despite these advances, proteomic data specifically characterizing canine KCS remain limited. Therefore, the present study was designed as an exploratory, hypothesis-generating pilot investigation aimed at characterizing qualitative and structural alterations in the tear proteome of dogs with naturally occurring KCS, rather than serving as a confirmatory or longitudinal biomarker validation study.

## 2. Materials and Methods

### 2.1. Animals and Diagnostic Criteria

Tear samples were obtained from 12 beagle dogs—5 clinically healthy dogs and 7 client-owned dogs diagnosed with KCS. To minimize breed-related variability in tear proteomic profiles, both the healthy control group and the KCS group were restricted to Beagles. This breed was selected because it is commonly used in ophthalmic research and allows controlled comparison by reducing genetic and anatomical heterogeneity.

Healthy controls were required to exhibit Schirmer tear test (STT) values ≥ 15 mm/min and no ocular abnormalities on slit-lamp biomicroscopy (SL-15, Kowa Optimed, Inc., Tokyo, Japan) [13]. Dogs with KCS were included if they presented with chronic conjunctivitis, mucopurulent discharge, and STT values ≤ 10 mm/min in at least one eye. None of the enrolled dogs received topical or systemic immunosuppressive treatment for at least two weeks prior to tear collection.

All procedures were performed in accordance with institutional animal care and use guidelines, and informed owner consent was obtained. No intermediate or borderline tear-deficient group was included, as this study focused on comparing clearly defined physiological extremes (clinically healthy eyes versus established KCS) to improve the resolution of structural proteomic differences using 2DE.

### 2.2. Tear Collection and Protein Concentration Measurement

Tears were collected using sterile STT strips (Ayumi Pharmaceutical Corporation, Tokyo, Japan), placed in the ventral conjunctival fornix for 1 min. The wetted portion of each strip was excised, immediately frozen at −80 °C, and later extracted in phosphate-buffered saline. Total protein concentration was determined spectrophotometrically using a NanoDrop ND-1000 (Thermo Fisher Scientific, Waltham, MA, USA; originally developed by NanoDrop Technologies, Wilmington, DE, USA) at 280 nm, following trichloroacetic acid (TCA) precipitation and resuspension in urea/thiourea buffer.

### 2.3. Two-Dimensional Electrophoresis (2DE)

Extracted proteins were separated using 2DE as per established protocols [14]. In brief, isoelectric focusing (IEF) was performed using 7 cm Immobiline DryStrips (pH 3–11 nonlinear; GE Healthcare, Chicago, IL, USA) in the first dimension, followed by SDS-PAGE in the second dimension [14,15]. Gels were silver-stained and scanned using a high-resolution flatbed scanner. Gel images were analyzed using PDQuest software version 7.3.1 (Bio-Rad Laboratories, Hercules, CA, USA) to generate a master gel and quantify normalized spot volumes. Spots with ≥4-fold differences in abundance between groups were considered differentially expressed. Relative intensity changes were independently verified using ImageJ software (Version 1.49) (NIH, Bethesda, MD, USA) [16].

### 2.4. In-Gel Digestion and Protein Identification

MALDI-TOF-based peptide mass fingerprinting (PMF) primarily provides qualitative protein identification rather than absolute quantification and is therefore best suited for detecting robust compositional shifts rather than subtle changes in protein abundance.

Differentially expressed protein spots were excised, destained, and subjected to in-gel tryptic digestion. The resulting tryptic peptide extracts were desalted and concentrated using ZipTip C18 tips (Millipore, Billerica, MA, USA) and then eluted. The eluates were then mixed with 2 µL of matrix solution (saturated α-cyano-4-hydroxycinnamic acid in 50% acetonitrile, 0.1% trifluoroacetic acid), deposited onto a MALDI target plate, and subjected to MALDI-TOF MS [17,18] on a Voyager-DE STR system (Applied Biosystems, Foster City, CA, USA) operating in reflector mode. Mass spectra were calibrated using a peptide calibration standard, and protein identification was performed by PMF against the Swiss-Prot and NCBInr databases (taxonomy: *Canis familiaris*) using the Mascot search engine (Matrix Science, London, UK). Identified proteins were accepted only when at least two unique peptides matched, sequence coverage exceeded 20%, and Mascot scores surpassed the significance threshold (*p* < 0.05). For proteins with borderline coverage (20–30%), additional verification was conducted by confirming agreement between observed and theoretical peptide masses and by evaluating biological plausibility within the established canine tear proteome.

### 2.5. Low-Molecular-Weight Peptide Profiling

To examine low-molecular-weight (LMW) peptides (<10 kDa) not well-resolved by conventional 2DE, neat tear extracts (without TCA precipitation) were desalted using ZipTip C18 tips (Millipore, Billerica, MA, USA) and analyzed by MALDI-TOF MS, with the instrument set in linear mode with an acquisition range of 500–10,000 Da. Spectral profiles were compared between groups, focusing on the presence or absence of characteristic peaks in the 4–6 kDa range. This range corresponds to proline-rich proteins and other LMW tear peptides that play critical roles in tear film stability and ocular surface protection.

### 2.6. Statistical Analysis

Total protein concentrations between groups were compared using the Mann–Whitney U test (GraphPad Prism 9.0, GraphPad Software, San Diego, CA, USA). Results are expressed as mean ± standard deviation (SD), with *p* < 0.05 considered statistically significant.

## 3. Results

### 3.1. Tear Production and Total Protein Concentration

Healthy beagles showed STT values of 22.9 ± 5.6 mm/min, whereas dogs with KCS exhibited STT values of 5.4 ± 1.8 mm/min, consistent with the diagnostic threshold used for inclusion. Total tear protein concentration, determined by UV absorbance at 280 nm was significantly higher in the KCS group (30.7 ± 13.5 mg/mL) than in the healthy controls (11.5 ± 1.8 mg/mL; Mann–Whitney U test, *p* < 0.032; Table 1).

Although the dogs in the KCS group exhibited significantly higher total tear protein concentration (30.7 ± 13.5 mg/mL) than healthy dogs (11.5 ± 1.8 mg/mL), their overall tear production rate was markedly reduced (5.4 ± 1.8 vs. 22.9 ± 5.6 mm/min).

When tear volume and protein concentration were integrated, the estimated total protein flux per minute in the KCS group was approximately 0.64 relative to that of the healthy controls (set as 1.00; Table 2). This indicates that overall tear protein output was reduced despite the elevated protein concentration. This pattern suggests that, although protein concentration increased—likely due to tear film dehydration and the presence of serum-derived proteins—the total secreted protein output was diminished in the KCS group, reflecting the combined effects of aqueous deficiency and compositional remodeling of the tear film.

Statistical testing was limited to total tear protein concentration and STT values, as this study was not designed for quantitative inter-protein comparison; Table 2 and Table 3, therefore, present descriptive and structural comparisons only.

### 3.2. Disease-Associated Protein Spot Changes Revealed by 2DE

**Figure 1:**
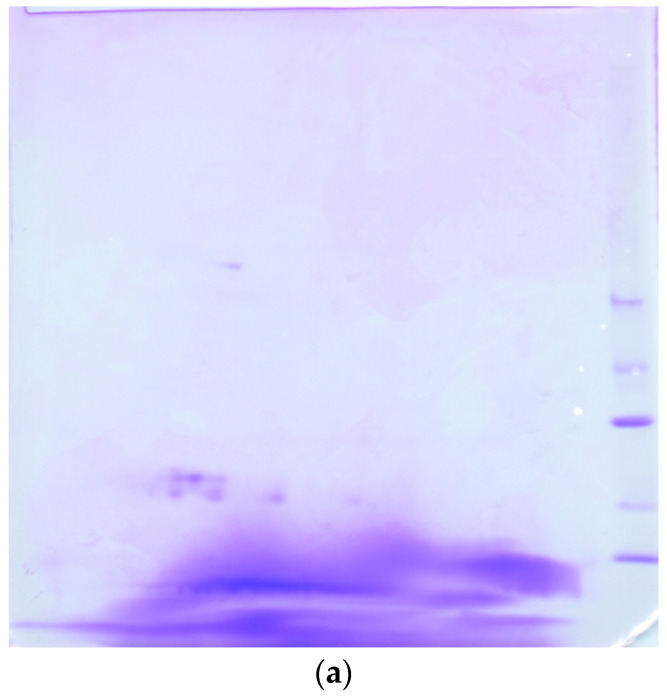

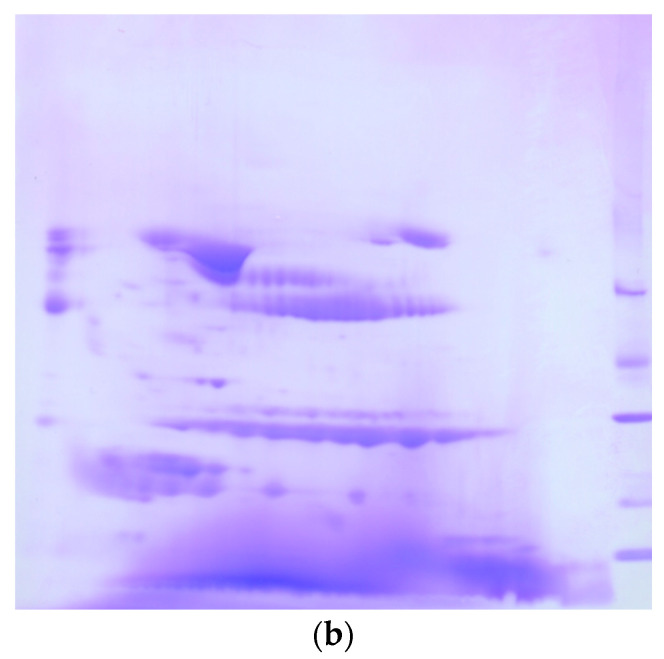
Two-dimensional electrophoresis (2DE) profiles of canine tear samples. (**a**) Representative 2DE map of tear samples obtained from a healthy dog. (**b**) A 2-DE map of tear samples from a dog with KCS, showing enrichment of high-molecular-weight proteins and reduction in low-molecular-weight peptides (<10 kDa) relative to the healthy control.

**Figure 2:**
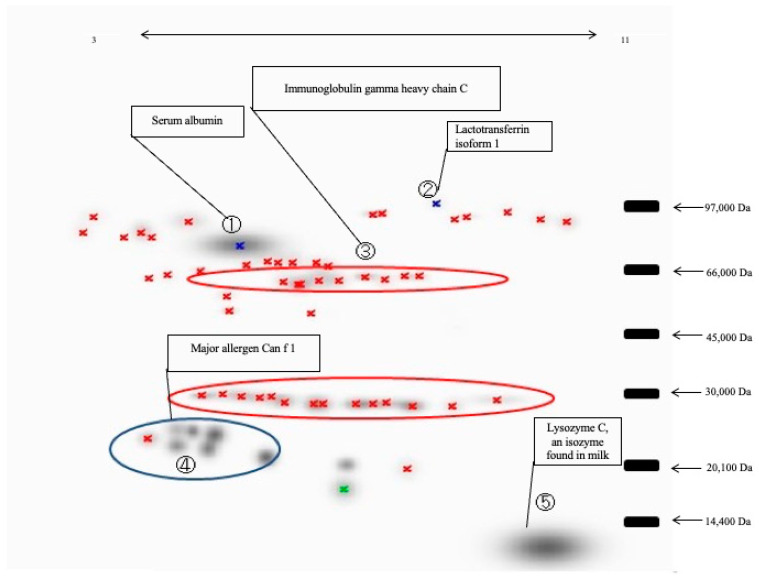
Master gel of tear proteins from dogs with KCS. The representative 2DE master gel was constructed from tear samples of dogs with KCS. The gel displays the reference protein spots used for intersample alignment and identification by MALDI-TOF MS. Protein spots with altered abundance compared with healthy controls were selected for PMF. The raw, uncropped gel images used to generate this composite master gel are provided in the Supplementary Materials (Original Gel Images.pdf). Blue crosses indicate protein spots showing a ≥4-fold difference in abundance in dogs with KCS compared with healthy controls, whereas red and green crosses indicate protein spots specifically detected in dogs with KCS. Numbers 1–5 indicate the protein spots identified by MALDI-TOF MS as follows: (1) serum albumin, (2) lactotransferrin isoform 1, (3) immunoglobulin gamma heavy chain C, (4) major allergen Can f 1, and (5) lysozyme C (milk isozyme).

**Figure 3:**
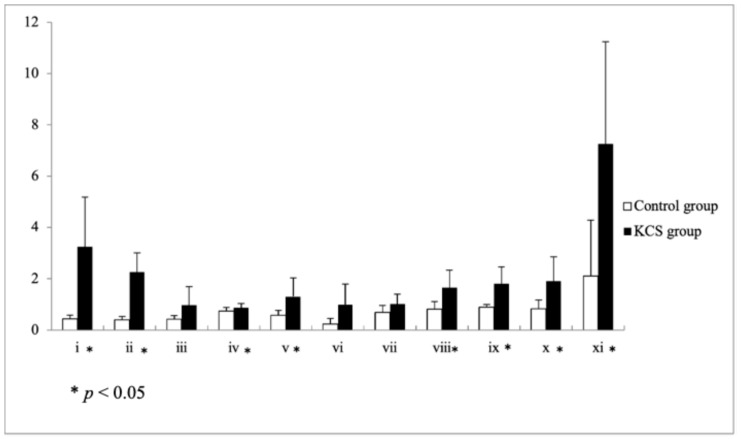
Quantitative comparison of 11 consensus protein spots between healthy and KCS-affected dogs. Bar graph representing the mean normalized intensities (±SD) of 11 consensus protein spots (i–xi) detected on the 2DE master gel. These spots were selected because they were consistently present in both groups, enabling direct cross-group comparison. Spots corresponding to high-molecular-weight proteins (e.g., i, ii, xi) showed increased abundance in the KCS group, whereas several low-molecular-weight spots (e.g., iv, v, viii, ix, x) exhibited markedly reduced intensity. These data confirm the bidirectional remodeling pattern identified in PDQuest analysis, with enrichment of high-molecular-weight components and selective depletion of low-molecular-weight peptides in KCS. * *p* < 0.05. Values represent normalized spot intensities expressed in arbitrary units (a.u.).

Figure 1 shows the discrete spot patterns of tear proteins from both the KCS and control groups, as resolved by 2DE. Image analysis using PDQuest software (version 7.3.1) generated a master gel incorporating all consistently detected spots (Figure 2). When the preset threshold of ≥4-fold difference in normalized spot volume was applied, multiple protein spots were found to differ between groups. Compared to the healthy controls, the KCS group exhibited increased intensity in several high-molecular-weight (HMW) spots and markedly reduced intensity in LMW spots. Quantitative cross-validation, carried out on 11 consensus spots (labeled i–xi on the master map) using ImageJ software (Version 1.49) (area × intensity as a proxy for abundance), confirmed the directional changes highlighted by PDQuest analysis (Figure 3).

The biological functions and major roles of the proteins identified by MALDI-TOF-MS are summarized in Table 4, highlighting their involvement in immune defense, antimicrobial activity, and tear film homeostasis.

### 3.3. Protein Identification by PMF

PMF of the mass spectra—obtained via MALDI-TOF MS of tryptic digests from differential spots excised from 2D gels—against Swiss-Prot/NCBInr (taxonomy: *Canis familiaris*) resulted in the identification of 5 proteins. These included serum albumin (coverage 37.0%; theoretical 68,605 Da/pI 5.5), lactotransferrin isoform 1 (43.6%; 77,296 Da/pI 8.6), immunoglobulin (Ig) gamma heavy chain C (25.3%; 51,843 Da/pI 6.2), major allergen Can f 1 (58.6%; 19,248 Da/pI 5.9), and lysozyme C, milk isozyme (36.4%; 14,472 Da/pI 8.6) (Table 3). Notably, albumin, lactotransferrin, and Ig heavy chain were up-regulated in KCS samples compared to the controls, aligning with the increased intensity of HMW spots observed on the 2DE gels (Figure 2).

### 3.4. LMW Peptide Profiling of Neat Tear Samples by MALDI-TOF MS

MALDI-TOF MS of desalted neat (pre-TCA) tear samples—carried out in linear mode (acquisition 500–10,000 Da) to identify small tear proteins and peptides—revealed prominent peaks at *m*/*z* 4400–4700 and *m*/*z* 5500–5750 in the healthy controls. However, these signals were markedly reduced or absent in the KCS samples. This selective loss of LMW signals in the KCS-affected dogs is congruent with the diminished staining observed in the lower gel regions and suggests depletion of proline-rich protein-like species and other small tear proteins during KCS.

### 3.5. Summary of Compositional Shifts

Integration of quantitative concentration data with 2D/PMF and LMW MALDI profiles revealed that tears from KCS-affected eyes are characterized by (i) increased overall protein concentration despite reduced aqueous flow, (ii) relative enrichment of HMW proteins (albumin, lactotransferrin, and Ig heavy chain), and (iii) reduction or loss of LMW proteins (<10 kDa), collectively indicating qualitative remodeling of the tear proteome in dogs affected by KCS.

## 4. Discussion

This study investigated the tear proteome of dogs affected by KCS and compared it with that of healthy controls. Five proteins were identified as differentially expressed: serum albumin, lactotransferrin isoform 1, Ig gamma heavy chain C, major allergen Can f 1, and lysozyme C. The biological functions of these differentially expressed proteins are summarized in Table 4, demonstrating a predominant enrichment of immune-related and antimicrobial components in dogs with KCS. In tear samples of the KCS-affected group, HMW proteins were upregulated, whereas LMW protein (<10 kDa, proline-rich protein-like) were reduced or completely absent. These findings suggest that both the composition and structural distribution of tear proteins are altered in dogs with KCS, suggesting that this disorder affects not only tear quantity but also its molecular quality.

The total tear protein concentration was significantly higher in the dogs with KCS (30.7 ± 13.5 mg/mL) than in the healthy dogs (11.5 ± 1.8 mg/mL). This pattern contrasts with findings pertaining to human nonautoimmune dry eye, where total protein levels have been shown to decrease. For instance, Versura et al. reported significantly lower total tear protein concentrations in patients with evaporative or age-related dry eye than in healthy individuals (9.89 ± 2.28 vs. 6.44 ± 2.1 mg/mL), while also identifying lysozyme-C (LYS-C), lactoferrin (LACTO), tear lipocalin-1 (LIPOC-1), zinc-alpha-2-glycoprotein (ZAG-2), transferrin (TRANSF), and serum albumin (ALB) as the predominant tear proteins [22].

The increased concentration of HMW proteins such as albumin and lactotransferrin in tear samples from KCS-affected dogs may reflect not only passive vascular leakage but also altered secretory dynamics within the lacrimal apparatus. Histochemical evidence has shown that the canine lacrimal gland consists of two morphologically distinct secretory cell types, namely acinar (type A) and tubular (type T) cells, which differ in their expression of Rab and SNARE family proteins responsible for the regulation of vesicular exocytosis [19]. This suggests that inflammatory or immune-mediated signaling during KCS selectively modulates vesicular transport pathways, thereby enhancing glandular secretion of plasma-related proteins such as lactoferrin and albumin. We refer to this proposed mechanism as the lacrimal secretory regulation hypothesis. In addition, chronic conjunctival inflammation may contribute to secondary introduction of locally produced or plasma-derived proteins into the tear film (postsecretory origin), as suggested by the increased vascular permeability and immune cell infiltration observed in the conjunctival epithelium of patients with DED. Both these mechanisms likely operate concurrently, reflecting a multifactorial alteration of the ocular surface proteome. Thus, the altered tear proteome can be viewed as a composite readout of concurrent changes in glandular secretion, local immune activity, and barrier integrity.

Previous proteomic investigations in healthy dogs have identified major canine allergen (MCA), serum albumin, the UPF0557 protein C10orf119 homolog, collagen alpha-2(I) chain, tyrosine-protein kinase Fer, keratin type II cytoskeletal protein, beta-crystallin B2, interleukin-6, and desmin as principal components of the canine tear film [8]. More recently, Winiarczyk et al. used MALDI-TOF MS to analyze tear samples from healthy male and female dogs and reported sex-related variations in tear protein composition [10]. While their work focused on physiological variations in healthy dogs, the present study furthers understanding in this field by highlighting disease-associated alterations in the tear proteome. In addition to confirming the presence of several previously reported proteins—including albumin, lactotransferrin, and lysozyme—in the tear proteome, we demonstrated that these proteins were differentially expressed during KCS. Notably, the increased abundance of albumin, lactotransferrin, and Ig heavy chain is indicative of an inflammatory response and heightened permeability of the ocular surface barrier [20]. These findings align with human studies where ALB, LACTO, and LIPOC-1 have been recognized as early indicators of ocular surface diseases [22]. It should be noted that protein identification via MALDI-TOF-based PMF is inherently qualitative and may yield limited sequence coverage for certain proteins. Even so, the concordance of peptide mass patterns, together with the biological plausibility of the detected proteins, supports the reliability of these identifications.

Evidence from human Sjögren’s syndrome further supports this interpretation. In this autoimmune condition, tear secretion is markedly reduced, yet normalized protein concentration increases as a result of inflammatory leakage. Li et al. reported a dramatic reduction in tear volume (2.13 ± 2.38 mm/5 min vs. 14.44 ± 6.57 mm/5 min in controls) accompanied by elevated protein levels [21]. Subsequent proteomic studies have confirmed extensive remodeling of the tear proteome during Sjögren’s syndrome, with hundreds of proteins differentially expressed and most showing upregulation [23,24]. Collectively, these findings highlight that reduced tear volume is tightly linked to compensatory proteomic remodeling—a mechanism that may likewise contribute to the tear film alterations observed in canine KCS.

Canine KCS shares important pathological features with human Sjögren’s syndrome, including progressive lymphocytic infiltration of the lacrimal glands, primarily by CD4+ T cells and B cells [2,25]. As in humans, the condition may occur in dogs as an isolated condition or in association with systemic autoimmune disorders such as diabetes mellitus or hypothyroidism [26]. In dogs, KCS carries additional clinical risks, including a high propensity for corneal ulceration and subsequent vision loss, underscoring the importance of early diagnosis and careful therapeutic monitoring in veterinary contexts. However, under-recognition of Sjögren’s syndrome remains a challenge even in human ophthalmology. A survey of U.S. ophthalmologists revealed that more than half refer fewer than 5% of their dry eye patients for systemic Sjögren’s evaluation, highlighting the continuing need for evidence-based referral criteria and standardized diagnostic pathways [27].

The enrichment of high-molecular-weight proteins such as albumin and Ig heavy chains likely reflects plasma leakage across inflamed lacrimal ducts and compromised epithelial tight junctions. Chronic lymphocytic and macrophage infiltration within the glandular tissue may further disrupt acinar cell integrity and reduce secretion of small, acinar-derived peptides. This structural remodeling can impair tear film stability and weaken antimicrobial defense mechanisms, thereby perpetuating ocular surface inflammation.

The present results are consistent with, yet distinct from, previous canine studies. Sussadee et al. analyzed tear samples from dogs with KCS using LC–MS/MS and reported upregulation of proteins such as heat shock protein beta-1, S100-A12, and keratin type II cytoskeletal 1 and 5, along with downregulation of lysozyme [28]. Partial normalization was observed after cyclosporine treatment. Unlike their quantitative proteomic approach, our study used 2DE to resolve tear proteins by molecular weight and isoelectric point, enabling a direct comparison of protein composition between healthy and KCS-affected dogs.

Although this study was not designed for quantitative proteomic comparison, the combination of 2DE and MALDI-TOF MS enabled structural visualization of molecular-weight-dependent remodeling of the tear proteome. This qualitative approach revealed a clear compositional shift, characterized by enrichment of HMW proteins and selective depletion of LMW peptides (<10 kDa). These changes provide structural evidence that KCS alters not only the total protein content but also the molecular architecture of the tear film. Similar compositional shifts have been reported in human dry eye, where inflammatory proteins such as S100A8 and S100A9 are elevated [29], whereas lipocalin-1, lipophilin, and other small tear proteins are reduced [30,31]. Collectively, these findings indicate that loss of LMW tear components may serve as an early marker of tear film instability and emerging ocular surface dysfunction.

Among the depleted LMW components, PRR4 warrants particular attention. PRR4 is a lacrimal-gland-derived secretory protein involved in mucosal protection, lubrication, and modulation of local immune responses. Reduced PRR4 expression has been reported in human dry eye and Sjögren’s syndrome, where acinar cell dysfunction diminishes synthesis and secretion of this peptide [6]. The observed loss of proline-rich protein-like signals in canine KCS may thus reflect a similar impairment of acinar secretory function. Such depletion could compromise tear film stability and antimicrobial defense, thereby contributing to ocular surface inflammation.

Overall, the findings indicate that KCS alters both the quantity and quality of tear proteins. The increased concentration of albumin and lactotransferrin may represent a protective response to ocular surface inflammation, while the loss of small peptides could weaken antimicrobial defense and tear film stability. Proteomic profiling may therefore be of value as a complementary diagnostic and monitoring tool, bolstering traditional assessments such as the Schirmer tear test.

While previous studies have identified individual inflammatory and structural proteins altered in canine KCS, the present work provides a complementary structural perspective by mapping the molecular-weight-dependent remodeling of the tear proteome. This approach highlights qualitative shifts in tear composition that may contribute to reduced tear film stability and compromised ocular surface protection.

## 5. Limitations

Several limitations of this study should be acknowledged. First, this investigation was designed as a pilot, exploratory study and did not include clinical severity indices, inflammatory biomarkers, treatment response, or longitudinal follow-up. Therefore, the present findings should be interpreted as structural proteomic observations rather than validated clinical biomarkers.

Second, the small sample size reflects both the labor-intensive nature of 2DE-based proteomic analysis and the difficulty of recruiting clinically confirmed KCS cases with comparable disease severity and uniform treatment history. Although statistical generalizability was not the objective of this study, the observed compositional shifts provide reproducible preliminary evidence of tear proteome remodeling associated with canine KCS. All dogs included in this study, including both healthy controls and dogs with keratoconjunctivitis sicca, were Beagles; therefore, breed-related confounding within the study population is unlikely. However, restriction to a single breed limits direct extrapolation of these findings to other breeds.

Third, the PMF approach used in this study is primarily qualitative, and subtle quantitative differences or low-abundance proteins may not be fully captured. In addition, the absence of LC–MS/MS or immunoblot validation limits definitive identification of some low-intensity spots. Future studies incorporating quantitative proteomic techniques, multi-breed cohorts, and longitudinal sampling will be necessary to validate these findings and to clarify the clinical significance of tear proteome alterations in KCS.

## 6. Conclusions

Preliminary proteomic analysis of tear protein samples revealed significant compositional alterations in dogs with KCS. These findings support the concept that, in clinical cases of KCS, not only the volume of tear secretion but also the qualitative integrity of the protective tear protein repertoire is altered. The observed upregulation of HMW proteins and the concomitant depletion of LMW components indicate structural remodeling of the tear proteome in association with ocular surface inflammation. Further studies incorporating larger sample sizes and quantitative proteomic platforms such as LC–MS/MS are warranted to validate these findings and to assess the potential of tear proteins as biomarkers for diagnosis and therapeutic monitoring in canine KCS.

## Figures and Tables

**Table 1:** Demographic and clinical information of enrolled dogs.

Dog ID	Group	Breed	Sex	Age (Years)	STT OD (mm/min)	STT OS (mm/min)
H1	Healthy	Beagle	F	4.8	35	27
H2	Healthy	Beagle	M	2.1	15	18
H3	Healthy	Beagle	M	2.1	23	25
H4	Healthy	Beagle	F	3	20	21
H5	Healthy	Beagle	F	3.1	20	25
	Healthy summary		M:2/F:3	2.8 (range 2.1–4.8)	22.6 (range 15–35)	23.2 (range 18–27)
K1	KCS	Beagle	F	4.7	3	3
K2	KCS	Beagle	F	10.5	3	—
K3	KCS	Beagle	F	7	6	—
K4	KCS	Beagle	M	9.5	—	7
K5	KCS	Beagle	F	4.4	7	12
K6	KCS	Beagle	F	4.7	7	5
K7	KCS	Beagle	M	9.3	12	7
	KCS summary		M:2/F:5	7.0 (range 4.4–10.5)	6.3 (range 3–12)	6.8 (range 3–12)
	*p* value			—	<0.001	<0.001

Note: All dogs were Beagles. “—” indicates that STT was not recorded in that eye. For each dog, a representative STT value was defined as the lower value of the two eyes when bilateral measurements were available. Group comparisons between healthy and KCS dogs were performed using the Mann–Whitney U test.

**Table 2:** Estimated tear protein flux calculated from STT-derived tear volume and protein concentration.

Parameter	Healthy Dogs (Mean ± SD) Dogs	Dogs with KCS (Mean ± SD)	Relative Ratio (KCS/Healthy)
Schirmer tear test (STT, mm/min)	22.9 ± 5.6	5.4 ± 1.8	0.24×
Protein concentration (mg/mL, UV method)	11.5 ± 1.8	30.7 ± 13.5	2.7×
Estimated total tear protein flux (volume × concentration, normalized)	—	—	0.64×

Note: Tear volume was estimated from per-dog representative Schirmer tear test (STT) values, and total protein concentration was determined by UV spectrophotometry. The estimated tear protein flux represents the relative amount of tear proteins secreted per minute and was calculated by multiplying relative tear volume by relative protein concentration. These values are intended for descriptive comparison only; no statistical analysis was performed, as this study was not designed for quantitative assessment of tear protein output. Although protein concentration increased during keratoconjunctivitis sicca (KCS), the markedly reduced tear volume resulted in a lower overall estimated protein output.

**Table 3:** Proteins identified by MALDI-TOF MS.

No.	Coverage (%)	TIC (%)	Mean Error (Da)	Data Tolerance (Da)	MS-Digest Index	Protein MW (Da/pI)	Accession No.	Protein Name
1	37.0	44.0	0.0289	0.2550	11,966	68,605/5.5	P49822	Serum albumin
2	43.6	43.0	0.0383	0.2420	1,983,882	77,296/8.6	Q9XSJ6 (canine lactotransferrin)	Lactotransferrin isoform 1
3	25.3	22.0	0.0986	0.3490	2,248,327	51,843/6.2	A0A5F4C7V2 (canine IgG heavy chain constant region)	Immunoglobulin gamma heavy chain C
4	58.6	26.0	0.1180	0.3350	118,387	19,248/5.9	O18873	Major allergen Can f 1
5	36.4	22.0	−0.0137	0.0223	129,505	14,472/8.6	P81708	Lysozyme C, milk isozyme

Note: Proteins were identified by PMF using MALDI-TOF MS. This table presents qualitative protein identification only; no quantitative comparison or statistical analysis was performed. Sequence coverage ranged from 25% to 59%. Although lower coverage (<30%) may reflect partial ionization or post-translational modifications, all identifications were supported by consistent theoretical peptide mass matching. Coverage (%) indicates the proportion of matched peptide sequences relative to the theoretical protein sequence. TIC (%) represents the relative total ion current. Protein molecular weight (MW) and isoelectric point (pI) were calculated based on the corresponding accession entries in the UniProt database.

**Table 4:** Biological functions of proteins identified in canine tear samples.

Protein	Function Category	Representative Functions	References
Serum albumin	Transport/Osmoregulation	Carrier protein; maintains osmotic pressure	[18]
Lactotransferrin isoform 1	Antimicrobial/Immune	Iron-binding; bacteriostatic	[19,20]
Ig gamma heavy chain C	Immune response	Major component of IgG	[20,21]
Lysozyme C	Antimicrobial	Hydrolyzes peptidoglycan	[18]
Can f 1	Allergens/Immune	Lipocalin family; antigenic protein	[8,10]

Note: This table summarizes the major functional categories and representative biological roles of tear proteins identified as differentially expressed in dogs with keratoconjunctivitis sicca. Functional annotations are based on established literature and UniProt database information.

## Data Availability

The original contributions presented in this study are included in the article/Supplementary Material. Further inquiries can be directed to the corresponding author.

## Supplementary Materials

The following supporting information can be downloaded at https://www.mdpi.com/article/10.3390/ani16020160/s1; The original, uncropped gel images used to generate the composite master gel are available as Supplementary Materials.

## References

[1] Stapleton F., Argueso P., Asbell P., Azar D., Bosworth C., Chen W., Ciolino J.B., Craig J.P., Gallar J., Galor A. (2025). TFOS DEWS III: Digest. Am. J. Ophthalmol..

[2] Gao J., Gelber-Schwalb T.A., Addeo J.V., Stern M.E. (1998). Apoptosis in the lacrimal gland and conjunctiva of dry eye dogs. Lacrimal Gland, Tear Film, and Dry Eye Syndromes 2; Advances in Experimental Medicine and Biology.

[3] Quimby F.W., Schwartz R.S., Poskitt T., Lewis R.M. (1979). A disorder of dogs resembling Sjogren’s syndrome. Clin. Immunol. Immunopathol..

[4] Jung J.H., Ji Y.W., Hwang H.S., Oh J.W., Kim H.C., Lee H.K., Kim K.P. (2017). Proteomic analysis of human lacrimal and tear fluid in dry eye disease. Sci. Rep..

[5] Dor M., Eperon S., Lalive P.H., Guex-Crosier Y., Hamedani M., Salvisberg C., Turck N. (2019). Investigation of the global protein content from healthy human tears. Exp. Eye Res..

[6] Perumal N., Funke S., Wolters D., Pfeiffer N., Grus F.H. (2015). Characterization of human reflex tear proteome reveals high expression of lacrimal proline-rich protein 4 (PRR4). Proteomics.

[7] Ma J.Y.W., Sze Y.H., Bian J.F., Lam T.C. (2021). Critical role of mass spectrometry proteomics in tear biomarker discovery for multifactorial ocular diseases (Review). Int. J. Mol. Med..

[8] Winiarczyk M., Winiarczyk D., Banach T., Adaszek L., Madany J., Mackiewicz J., Pietras-Ozga D., Winiarczyk S. (2015). Dog tear film proteome in-depth analysis. PLoS ONE.

[9] Graham K.L., Diefenbach E., McCowan C.I., White A.J.R. (2020). A technique for shotgun proteomic analysis of the precorneal tear film in dogs with naturally occurring primary glaucoma. Vet. Ophthalmol..

[10] Winiarczyk D., Winiarczyk M., Michalak K. (2025). Proteomic analysis of tear films in healthy female and male dogs using MALDI-TOF (matrix assisted laser desortion/ionization time-of-flight) mass spectrometry. Animals.

[11] Winiarczyk D., Winiarczyk M., Balicki I., Szadkowski M., Michalak K., Winiarczyk S., Adaszek L. (2022). Proteomic analysis of tear film in canine diabetic patients with and without retinopathy. J. Vet. Res..

[12] Spitznagel K.M., Mikeska R., Jost H., McGrath S., Mehaffy C., Henriksen M.L. (2023). Detection of pro-inflammatory cytokines in healthy canine tears using Canine Cytokine SpikeMix mass spectrometry via multiple reaction monitoring. Vet. Ophthalmol..

[13] Giuliano E.A., Gelatt K.N. (2021). Diseases and surgery of the canine lacrimal secretory system. Vet Ophthalmol.

[14] Görg A., Obermaier C., Boguth G., Harder A., Scheibe B., Wildgruber R., Weiss W. (2000). The current state of two-dimensional electrophoresis with immobilized pH gradients. Electrophoresis.

[15] Görg A., Klaus A., Lück C., Weiland F., Weiss W. (2007). Two-Dimensional Electrophoresis with Immobilized PH Gradients for Proteome Analysis: A Laboratory Manual.

[16] Schneider C.A., Rasband W.S., Eliceiri K.W. (2012). NIH Image to ImageJ: 25 years of image analysis. Nat. Methods.

[17] Pappin D.J., Højrup P., Bleasby A.J. (1993). Rapid identification of proteins by peptide-mass fingerprinting. Curr. Biol..

[18] Henzel W.J., Billeci T.M., Stults J.T., Wong S.C., Grimley C., Watanabe C. (1993). Identifying proteins from two-dimensional gels by molecular mass searching of peptide fragments in protein sequence databases. Proc. Natl. Acad. Sci. USA.

[19] Yasui T., Miyata K., Nakatsuka C., Tsukise A., Gomi H. (2021). Morphological and histochemical characterization of the secretory epithelium in the canine lacrimal gland. Eur. J. Histochem..

[20] Flanagan J., Willcox M. (2009). Role of lactoferrin in the tear film. Biochimie.

[21] Li B., Sheng M., Li J., Yan G., Lin A., Li M., Wang W., Chen Y. (2014). Tear proteomic analysis of Sjogren syndrome patients with dry eye syndrome by two-dimensional-nano-liquid chromatography coupled with tandem mass spectrometry. Sci. Rep..

[22] Versura P., Bavelloni A., Grillini M., Fresina M., Campos E. (2013). Diagnostic performance of a tear protein panel in early dry eye. Mol. Vis..

[23] Yoon S.P., Yu Z., Pflugfelder S.C., de Paiva C.S. (2023). Differentially expressed tear proteins in Sjögren’s syndrome keratoconjunctivitis sicca. Transl. Vis. Sci. Technol..

[24] Kuo M.T., Fang P.C., Chao T.L., Chen A., Lai Y.H., Huang Y.T., Tseng C.Y. (2019). Tear proteomics approach to monitoring Sjögren’s syndrome or dry eye disease. Int. J. Mol. Sci..

[25] Williams D.L. (2008). Immunopathogenesis of keratoconjunctivitis sicca in the dog. Vet. Clin. N. Am. Small Anim. Pract..

[26] Kaswan R., Pappas C., Wall K., Hirsh S.G. (1998). Survey of canine tear deficiency in veterinary practice. Lacrimal Gland, Tear Film, and Dry Eye Syndromes 2.

[27] Bunya V.Y., Fernandez K.B., Ying G.S., Massaro-Giordano M., Macchi I., Sulewski M.E., Hammersmith K.M., Nagra P.K., Rapuano C.J., Orlin S.E. (2018). Survey of ophthalmologists regarding practice patterns for dry eye and Sjögren’s syndrome. Eye Contact Lens.

[28] Sussadee M., Rucksaken R., Havanapan P.O., Reamtong O., Thayananuphat A. (2021). Changes in tear protein profile in dogs with keratoconjunctivitis sicca following topical treatment using cyclosporine A. Vet. World.

[29] Zhou L., Beuerman R.W., Chan C.M., Zhao S.Z., Li X.R., Yang H., Tong L., Liu S., Stern M.E., Tan D. (2009). Identification of tear fluid biomarkers in dry eye syndrome using iTRAQ quantitative proteomics. J. Proteome Res..

[30] Versura P., Nanni P., Bavelloni A., Blalock W.L., Piazzi M., Roda A., Campos E.C. (2010). Tear proteomics in evaporative dry eye disease. Eye.

[31] Ham B.M., Jacob J.T., Cole R.B. (2007). Single-eye analysis and contralateral-eye comparison of tear proteins in normal and dry eye model rabbits by MALDI-ToF mass spectrometry using wax-coated target plates. Anal. Bioanal. Chem..

