# Supplementary material for: Tear Protein Alteration in Dogs with Keratoconjunctivitis Sicca
Source: Animals (Basel). 2026 Jan 6;16(2):160. doi: 10.3390/ani16020160 (PMC12837657; doi:10.3390/ani16020160)
Supplement: Supplementary file 1 [file animals-16-00160-s001.zip › animals-4018029-supplementary.pdf]

Original Gel Images

Healthy dogs raw 2DE gels

Healthy-1 (raw 2DE gel; pH 3–11 NL, 7 cm strip)

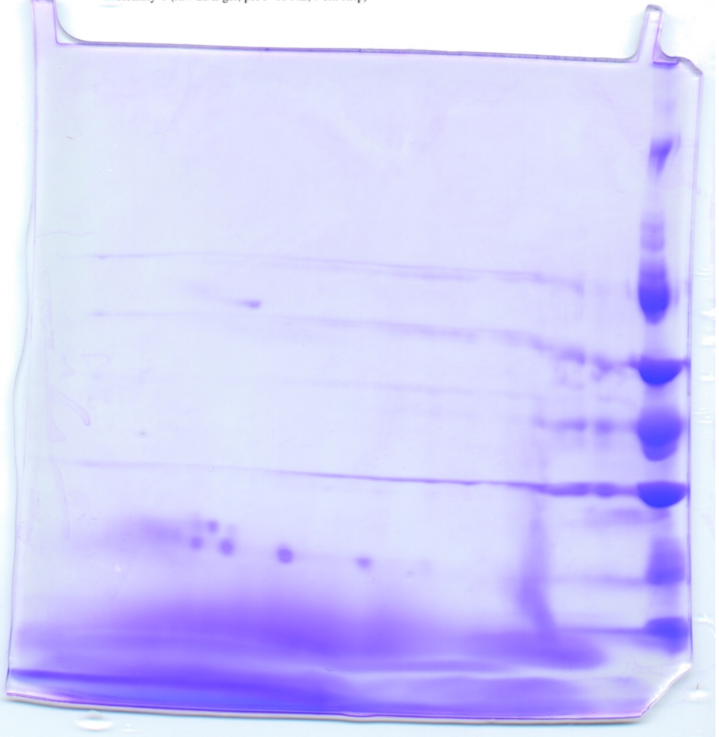

Healthy-2 (raw 2DE gel; pH 3–11 NL, 7 cm strip)

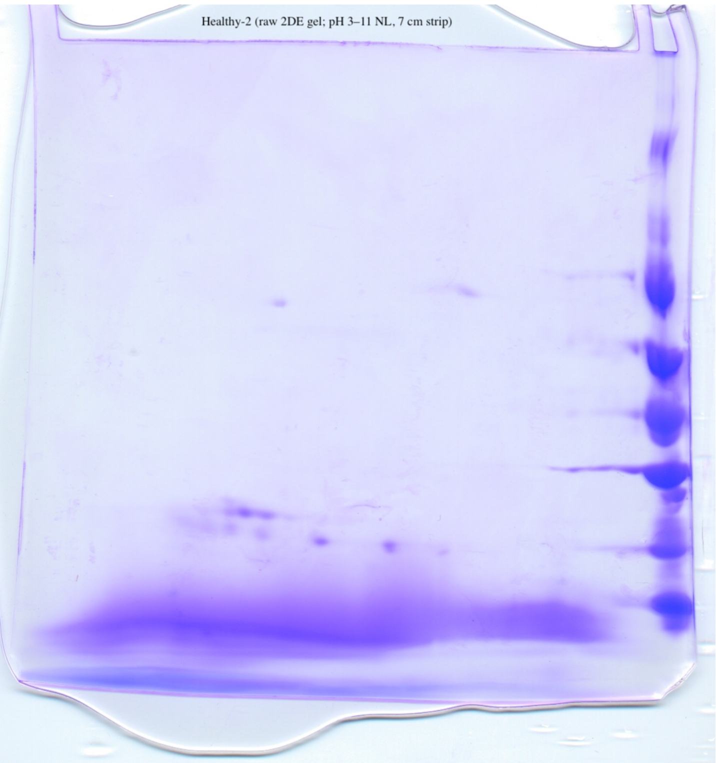

Healthy-3 (raw 2DE gel; pH 3–11 NL, 7 cm strip)

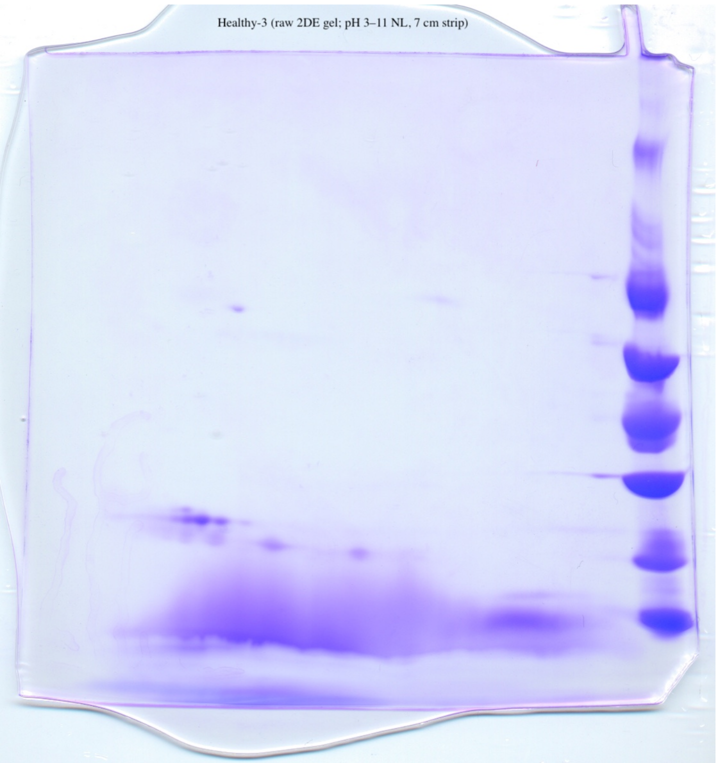

Healthy-4 (raw 2DE gel; pH 3–11 NL, 7 cm strip)

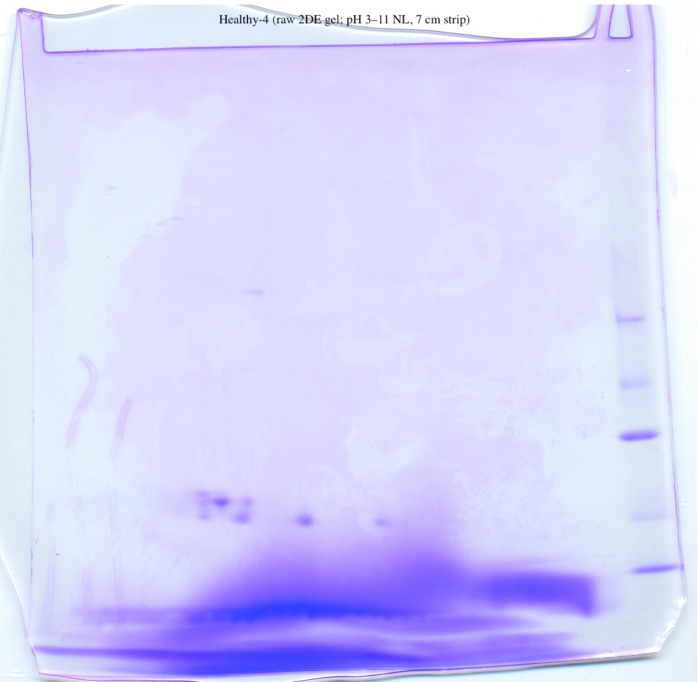

Healthy-5 (raw 2DE gel; pH 3–11 NL, 7 cm strip)

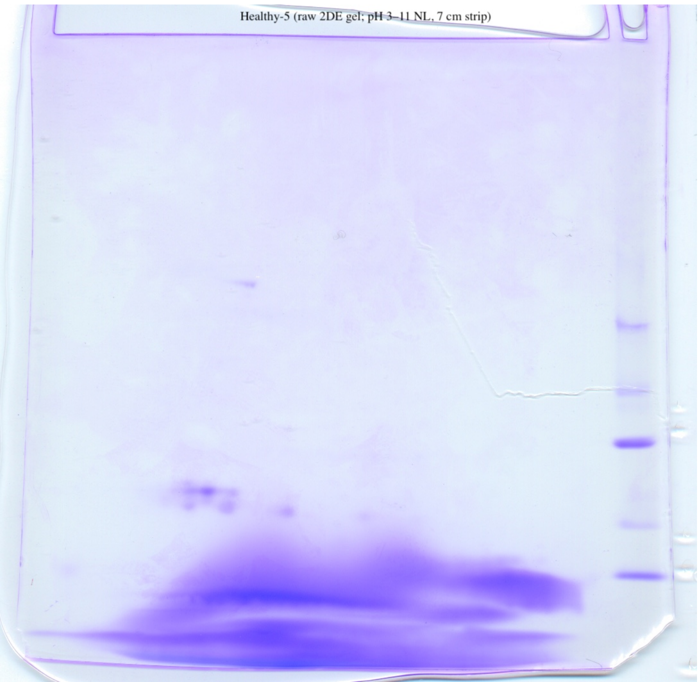

KCS dogs raw 2DE gels

KCS-1 (raw 2DE gel; pH 3–11 NL, 7 cm strip)

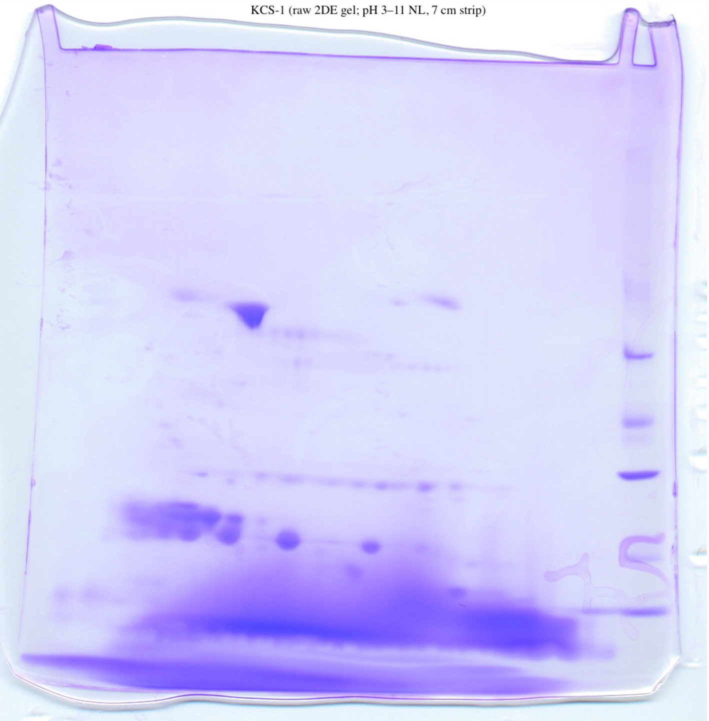

KCS-2 (raw 2DE gel; pH 3–11 NL, 7 cm strip)

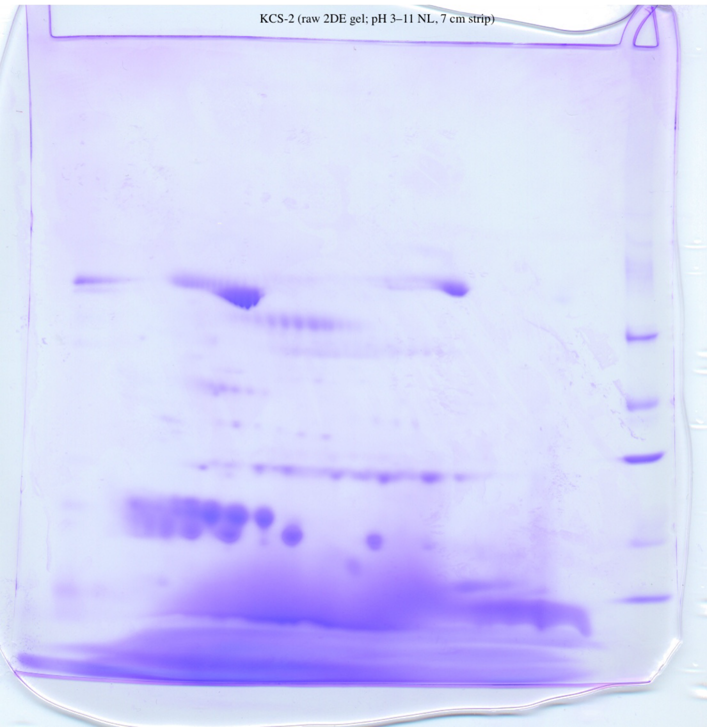

KCS-3 (raw 2DE gel; pH 3–11 NL, 7 cm strip)

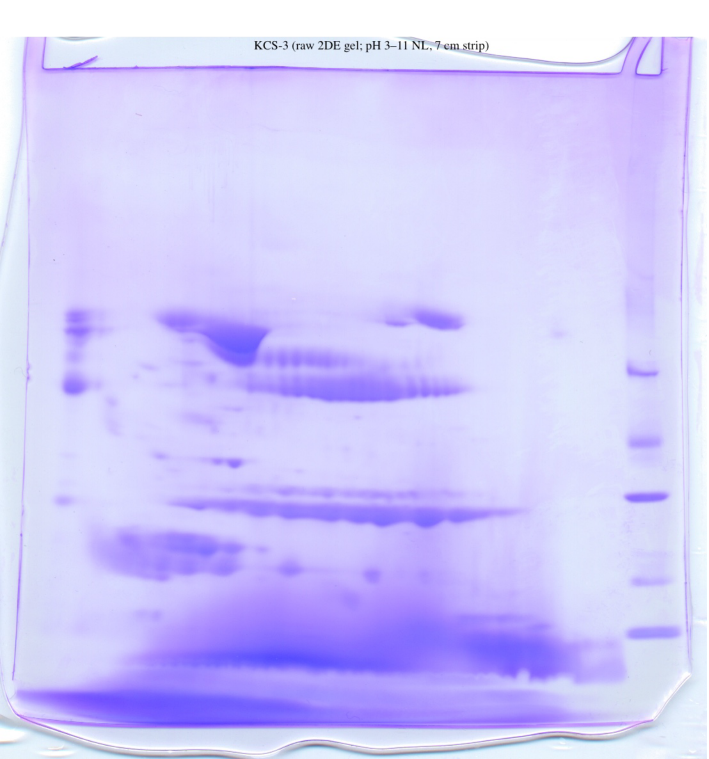

KCS-4 (raw 2DE gel; pH 3–11 NL, 7 cm strip)

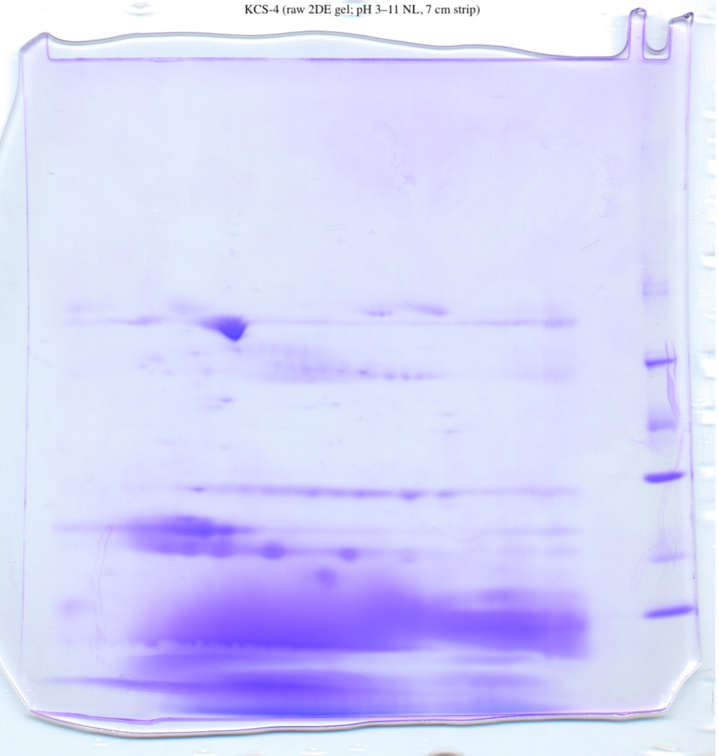

KCS-5 (raw 2DE gel; pH 3–11 NL, 7 cm strip)

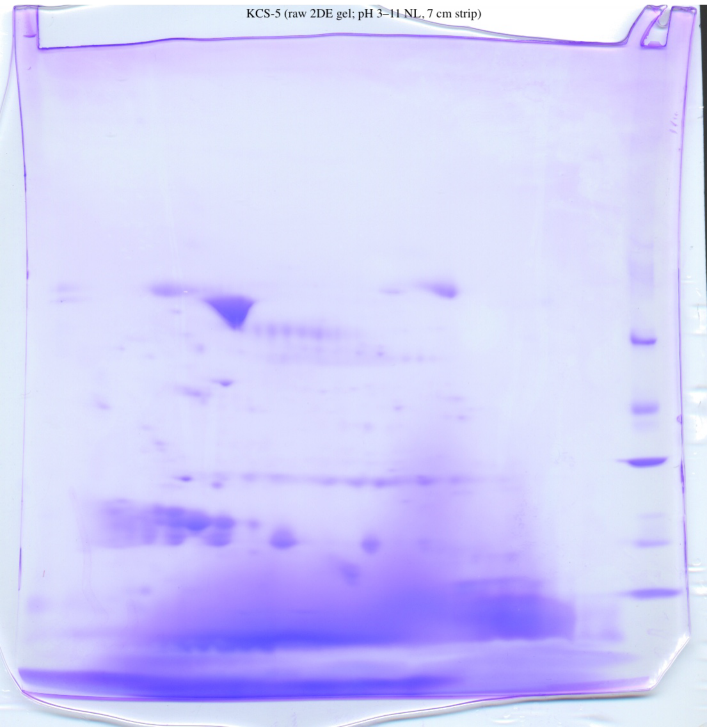

KCS-6 (raw 2DE gel; pH 3–11 NL, 7 cm strip)

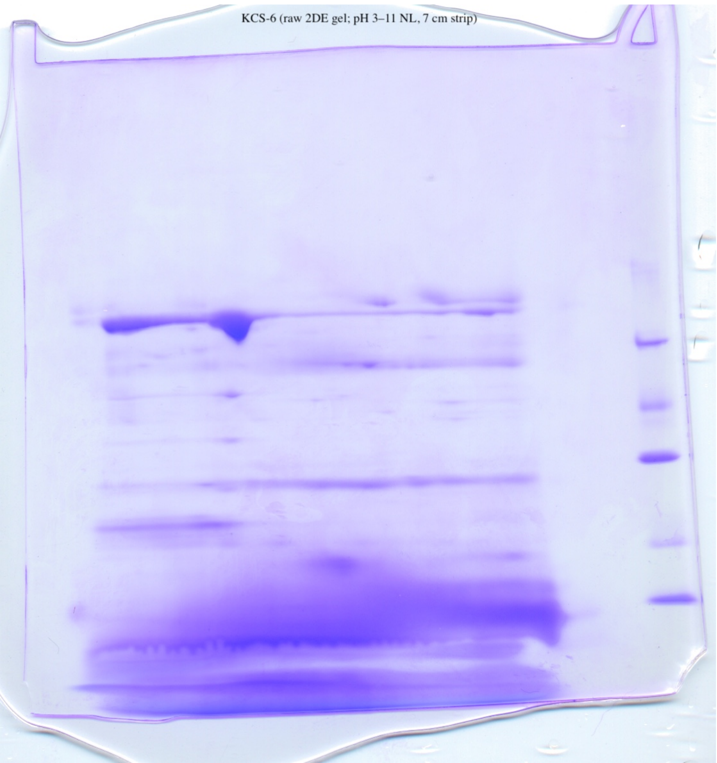

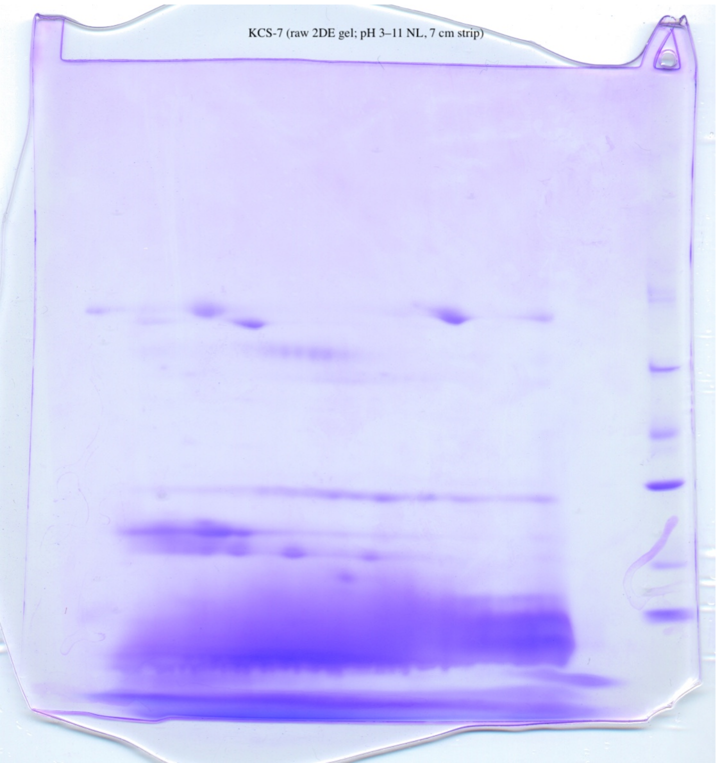
KCS-7 (raw 2DE gel; pH 3–11 NL, 7 cm strip)
